# Inhaled ceftazidime and amikacin versus inhaled colistin in the treatment of gram negative ventilator associated pneumonia

**DOI:** 10.1186/2197-425X-3-S1-A380

**Published:** 2015-10-01

**Authors:** YS Nassar, M Ibrahim, AG Salman, TS Elgohary

**Affiliations:** Cairo University, Cairo, Egypt

## Introduction

Aerosol antibiotics administration offers the theoretical advantages of achieving high drug concentrations at the infection site and low systemic absorption.

## Objectives

Comparing the microbiological outcome of inhaled ceftazidime amikacin versus inhaled colistin as adjunctives to conventional iv antibiotics in treating gram negative VAP.

## Methods

This prospective randomized controlled study was carried out on 60 mechanically ventilated patients with gram negative VAP.

***Inclusion criteria:*** Adult mechanically ventilated patients diagnosed to have Gram negative VAP confirmed:· Radiologically (new chest x- ray infiltrate).

· Microbiologically (positive culture from aspirate of endo- tracheal tube (ETT).

· Clinically (fever, leukocytosis and increased bronchial secretion). CPIS scoring >6.

**E*xclusion criteria:***· Severe renal impairment,

The patients included in the study were equally randomized to enter one of 3 groups; group A, group B or group C.

· **Group (A)** 20 patients: nebulized ceftazidime (15 mg.kg. 3 h) plus nebulized amikacin (25 mg·kg·d) in addition to conventional IV antibiotics.

· **Group (B)** 20 patients: nebulized Colistin (1million IU every 8h) in addition to

conventional IV antibiotics.

· **Group (C)** 20 patients: conventional IV antibiotics.

In all groups A,B and C, treatment was continued for five days followed by ETT aspirate.

Interpretation of culture results:

*Clearance:* no growth. *Resistance:* persistent pathogen. *Super infection:* eradication of previous pathogen with developing of another pathogen. *Resistance and super infection*: persistent responsible pathogen with developing of another pathogen.

## Results

The clearance of organism was (75% vs. 80% vs. 50%), resistance was (5% vs. 5% vs. 20%), superinfection was (0% vs. 10% vs. 15%), while combined resistance and super infection was (20% vs. 5% vs. 15%) in group A vs. B vs. C respectively.

***Comparing group B vs. C***: a significantly greater clearance (80% vs. 50%, **p 0.047**) while no significant difference regarding resistance (5% vs. 20%, p 0.151), superinfection (2% vs. 15%, p 0.633), combined resistance and super infection (5% vs. 15%, p 0.292) in group B vs. C respectively.

***Comparing group A vs. C***: no significant difference in clearance (75% vs.50%, p 0.102), resistance (5% vs. 20%, p 0.151), superinfection (0% vs. 15%, p 0.072), combined resistance and super infection (20% vs. 15%, p 0.667) in group A vs. C respectively.

***Comparing group A vs. B***: no significant difference in clearance (75% vs.80%, p 0.705), resistance (5% vs. 5%, p 1.0), superinfection (0% vs. 10%, p 0.147), combined resistance and super infection (20% vs. 5%, p 0.151) in group A vs. B respectively.

## Conclusions

The addition of Inhaled Colistin showed a significantly better organism clearance after 5 days compared to inhaled Ceftazidime and Amikacin and compared to iv antibiotics without additional inhaled antibiotics, in treating gram negative VAP.

## Grant Acknowledgment

Cairo University

